# Elder psychological abuse: narrative review of evidence and gaps in research

**DOI:** 10.3389/fpubh.2025.1694657

**Published:** 2025-10-31

**Authors:** Claudia Casella, Mariagrazia Marisei, Lucia Gloria, Maria Carrella, Gaetano Di Donna, Pierpaolo Di Lorenzo

**Affiliations:** Department of Advanced Biomedical Science-Legal Medicine Section, University of Naples “Federico II”, Naples, Italy

**Keywords:** elder abuse, emotional abuse, psychological abuse, caregivers, verbal abuse

## Abstract

**Introduction:**

Elder abuse is a substantial global public health and human rights problem, certainly one of the lesser known and studied forms of violence whose impact must not be overlooked because of its significant representation worldwide. Although psychological abuse has been found to be the most frequent form of abuse, specific knowledge regarding this form of violence is still lacking.

**Materials and methods:**

The PubMed database was searched using a combination of search terms related to older people, emotional and psychological violence and abuse and exclusion, as much as possible, of physical abuse.

**Results:**

Ten studies met inclusion criteria, representing diverse geographical contexts (United States, Poland, Taiwan) and settings (community, institutional). The analysis of the articles showed the absence of a shared definition, lack of structural conceptualization and extreme variability in the prevalence of the phenomenon reflecting the variability of definitions and socio-cultural heterogeneity and reduced reporting rates.

**Discussion and conclusions:**

The available literature on Elder Psychological Abuse is still limited and fragmented, with a small number of studies dedicated to this specific topic, the absence of a shared definition and the lack of standardized categorization and assessment tools. These elements underscore the need for further research to explore the phenomenon in depth, promoting the development of a single definition and reliable tools for its identification and classification.

## 1 Introduction

The World Health Organization defines elder abuse as a single or repeated act, or lack of appropriate action, occurring within a relationship of trust, that causes harm or distress to an older person.

Elder abuse is a substantial global public health and human rights problem. The World Health Organization has declared that elder abuse is a violation of one of a human being’s most basic fundamental rights, that of older persons to be safe and free of violence (1).

Although violence against older people is certainly one of the lesser-known and studied forms of violence, its impact must not be overlooked (2, 3) because of its significant representation worldwide.

In fact, the “World Report on Aging and Health” in 2015 (4) showed a prevalence of elder abuse ranging between 2.2 and 14%, including physical abuse (0.2–4.9%), sexual abuse (0.04–0.82%), psychological abuse (0.7–6.3%), financial abuse (1–9.2%) and neglect (0.2–5.5%), even though complete data regarding the most vulnerable older population (i.e., people affected by dementia and residents of nursing homes) are still lacking. This type of violence, such as genderbased violence, is still more frequent in women (5–7) and is less socially debated than other forms of abuse, with a little increase in attention only during the COVID-19 pandemic (8).

The known main risk factors for any kind of elder abuse are considered to be female sex, age > 74 years, disability and mental disorders, poor socioeconomic status, and social isolation. Today, the most common types of violence are neglect and psychological or emotional abuse, creating a picture that is clearly different from the past, dominated by physical and sexual violence (9, 10).

To this day, elder mistreatment is still a complex and only partially understood phenomenon, with numerous authors that, throughout the years, have tried to produce theories and models.

More specifically, within the wider categories of intrapersonal, interpersonal, sociocultural and multisystemic theory, authors like Pillemer and Wold (11), Wiber and McNeilly (12), Mosqueda et al. (13, 14) and Momtaz et al. (15) have hypothesized up to 13 different theories: caregiver stress theory, which posits that elder abuse occurs when a stressed/overburdened caregiver unleashes his/her frustrations on the care recipient. Stress can emerge either from personal factors (e.g., inadequate coping skills, multiple roles in the family, health problems, lack of caregiving skills), care-recipient factors (such as high levels of dependency, poor health, decreased mental capabilities or environmental factors), economic difficulties, lack of support from society-level agencies and social isolation. These factors combined can make the caregiver feel overburdened and frustrated, unleashing it on the care recipient. This theory has been controversial for several reasons, but mainly because it can be used as a strategy to blame the victim for the abuse, thus reducing the perpetrator’s accountability; social learning theory, which proposes that violence is learned through observation and modeled into our behavioral repertoire; bidirectional theory, which states that people raised in environments where violence is used as an interaction strategy or in situations where a caregiver or care receiver feels highly stressed are prone to violent outbursts, to which they are responded with more violence; psychopathology of the caregiver, which states that elder abuse emerges because the person assuming the caregiving role is suffering some form of psychopathology that makes him/her unable to provide adequate care or even prone to violence; social exchange theory, which posits that if one of the exchange partners has limited resources to trade and has increased needs, he/she will become “dependent” on his/her partner. In turn, this partner will gain more “power” over the relationship and manipulate the exchanges to maximize profit and cut losses.; dyadic discord theory statesthat conflict and discord emerge in a relationship because of contextual factors (history of family violence) and situational factors (e.g., low satisfaction with the relationship) and this discord might work as the onset for violence; power and control/feminist approach; ecological model, based on evidence that no single factor can explain why some people or groups are at higher risk of interpersonal violence, while others are more protected from it. This framework views interpersonal violence as the outcome of interaction among many factors at four levels: individual (personal history and biological factors influence how individuals behave and increase their likelihood of becoming a victim or a perpetrator of violence, such s being victim of child maltreatment); personal relationships (such as family, friends, intimate partners and peers may influence the risks of becoming a victim or perpetrator of violence); community (contexts in which social relationships occur, such as schools, neighborhoods and workplaces, also influence violence); societal (factors like economic and social policies that maintain socioeconomic inequalities between people, the availability of weapons, and social and cultural norms such as those around male dominance over women, parental dominance over children and cultural norms that endorse violence as an acceptable method to resolve conflicts). The ecological framework helps explain the result—violence later in life—as the interaction of an individual risk factor, the consequences of complications during birth, and a relationship risk factor, the experience of poor parenting. This framework is also useful to identify and cluster intervention strategies based on the ecological level in which they act (16); sociocultural model; political-economic theory; role accumulation theory; stratification theory and symbolic interactionism.

In this complex picture, some authors (17) have shown how the multiple theories, often not completely accepted, seem to actually coexist and all have some kind of role in explaining older people abuse, just from different points of view.

That said, all these theories tend to explain older people abuse in general and often do not further analyze specific types of abuse (18), with the unspoken assumption that the mechanisms underlying different forms of abuse are always the same, often focusing only on physical function or physical dependency.

In the broader context of elder mistreatment, although, psychological abuse has been found to be the most frequent form of abuse, with specific knowledge regarding this form of violencestill lacking.

It encompasses behaviors such as humiliation, intimidation, isolation, verbal aggression, and disrespect. Yet the terminology varies, with “psychological,” “emotional” and “verbal” abuse often used interchangeably.

Therefore, the aim of this paper is to explore the quantity and quality of knowledge concerning the phenomenon of elder psychological abuse (EPA) within the medical scientific literature and to identify potential lacks in its understanding, highlighting general key features and characteristics of victims and abusers, to provide theoretical and practical information useful for policymakers in the identification of potential prevention strategies.

## 2 Materials and methods

To pursue the aim abovementioned, the authors carried out a narrative review following methodological guidance from Sukhera (19), Ferrari (20) and Grant & Booth (21). Narrative reviews are suited for fields where evidence is scarce and heterogeneous.

More specifically, this paper tries to answer the following research questions:

(1) How do definitional variations in elder psychological abuse reflect underlying theoretical and disciplinary perspectives?
(2) What patterns emerge in the conceptualization and measurement of elder psychological abuse across different cultural and institutional contexts?
(3) Are there known features of victims and perpetrators of elder psychological abuse?
(4) How do current conceptualization and classification explain the complexity and heterogeneity observed in elder psychological abuse research?

To this purpose, given the medical extraction of the Authors, a choice was made to explore only the PubMed database, using a combination of search terms related to older people, psychological violence and abuse and exclusion, as much as possible, of search terms related to physical abuse, trying to yield the largest number of scientific articles concerning all forms of violence other than physical.

The choice to exclude other more interdisciplinary databases such as PsycINFO or CINAHL came from the acknowledgment that the lack of the Authors’ expertise in sociological, ecological and psychological fields could have potentially produced biases in the interpretation of data extracted from said search engines.

A search string was developed as follows: (“psychological abuse”[Title/Abstract] OR “psychological violence”[Title/Abstract] OR “emotional abuse”[Title/Abstract] OR “emotional violence”[Title/Abstract]) AND (“elder abuse”[Title/Abstract] OR “elder mistreatment”[Title/Abstract] OR “elder neglect”[Title/Abstract] OR “older adults”[Title/Abstract] OR “older people”[Title/Abstract]) NOT (“physical abuse”[Title/Abstract] OR “physical violence”[Title/Abstract] OR “physical maltreatment”[Title/Abstract] OR “bodily harm”[Title/Abstract]) AND (“humans”[MeSH Terms]) AND (“english”[Language]).

There were no limitations regarding publication date or geographic areas.

Inclusion criteria were the use of the English language and the presence of an explicit analysis of key features of various forms of psychological abuse, such as a specific definition, prevalence and theoretical conceptualization of the phenomenon.

Exclusion criteria were the use of languages different than English and the presence of data or explicit analysis of forms of physical violence or other forms of abuse other than psychological.

Title and abstract screening were carried out by two independent reviewers for assessment against the inclusion and exclusion criteria for the review, followed by full-text screening of selected citations. The Preferred Reporting Items for Systematic Reviews and Meta-Analyses extension for Scoping Reviews (PRISMA-ScR) guided the selection process and the reporting of results (22).

**Figure 1:**
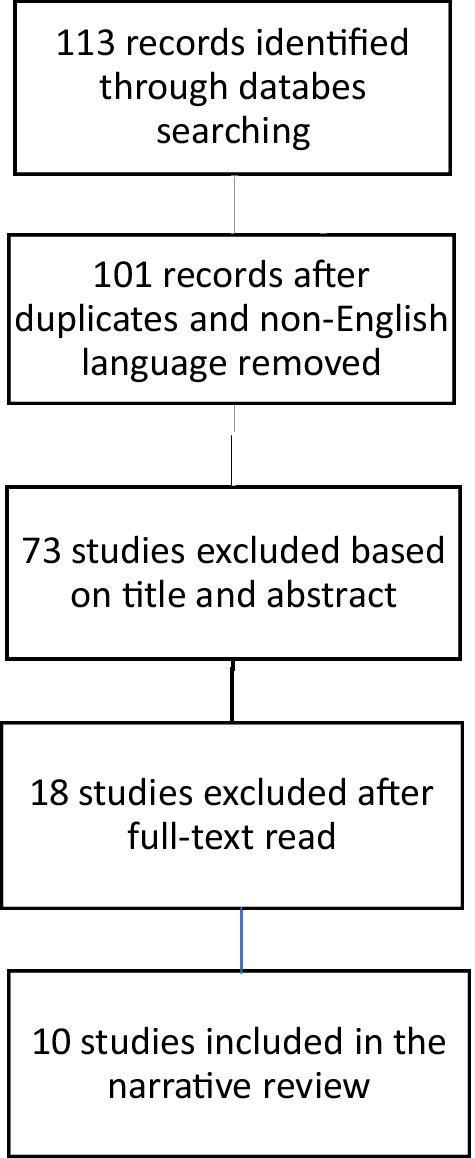
Flow diagram of studies identified in the systematic review.

One hundred and thirteen articles were identified from the selected database. Twelve duplicates and articles not written in English were excluded. Seventy-three manuscripts were excluded after screening the titles and abstracts. Of the 28 studies assessed for full-text eligibility, 18 were excluded. Ten articles were finally included in this narrative review (Figure 1), published from 2006 to 2025.

## 3 Results

The key findings are summarized in Table 1.

**Table 1:** Summary of included studies.

Author/ Year	Country	Sample	Definition	Instrument	Prevalence	Key findings
Fulmer et al., 2014 (22)	USA	Older adults enrolled from a large urban medical practice and academic dental practice in a large, diverse metropolitan setting	(a) intentional actions that cause harm or create a serious risk of harm (whether or not harm is intended) to a vulnerable elder by a caregiver or other person who stands in a trust relationship to the elder or (b) failure by a caregiver to satisfy the elder’s basic needs or to protect the elder from harm	CTS	38%	Older adults reporting verbal abuse also reported higher levels of depression and poorer quality of life compared to elderly individuals reporting no verbal abuse.
Filipska et al., 2020 (24)	Poland	Hospitalized elders	-	16-items custom questionnaire	29%	Risk factors for psychological abuse are female gender, age >70 years old, chronically illness, lower income, living in urban territories. Most frequent forms are arrogance, vulgarity and blackmail
Acierno et al., 2020 (25)	USA	Random older people over 68 years old	-	Interviews via random digit dialing methodology	8.4%	Almost 90% of financial and emotional forms of elder mistreatment by family, friend, or acquaintance was not reported, versus 33% of that perpetrated by strangers. Rates of non-reporting of emotional mistreatment at the hands of either strangers or family/friends/acquaintances was also about 85 to 90%
Chung et al., 2025 (26)	S. Korea	Community older adults	A form of abuse that involves the use of words, actions, or behaviors that cause psychological harm to an individual.	Custom survey	4.4%	Emotional abuse is predictive of suicidal ideation and depression
Wang JJ, 2006 (27)	Taiwan	Mixed (50.8% institutionalized and 49.2% domestic older adults partially dependent on a caregiver)	Inflicts anguish, pain, or distress through verbal aggression, threats, intimidation, insults, humiliation, and harassment, and can be both intentional or unintentional	PEAS, SPMSQ	22.6%	The domestic subjects exhibited a higher mean score of level of psychological abuse. Individuals with chronic diseases experienced more psychological abuse from their caregivers.
Conrad et al., 2011 (28)	USA	Mixed (elder abuse staff members and clients suspected of being victim of EPA)	Infliction of anguish, pain, or distress through verbal or nonverbal acts	OAPAM	43%	Isolation is the most serious type of EPA. Data supportive of the validity of OAPAM in helping to assess the existence and the level of EPA.
Conrad et al., 2011 (29)	USA	-	The infliction of anguish, pain, or distress through verbal or non verbal acts.	Concept mapping	-	Five clusters and two conceptual regions identified
Liu et al., 2019 (30)	USA	Alleged psychological abuse cases	The infliction of anguish, mental pain, or distress through verbal or non verbal acts	EADSS	-	Impaired cognition and increased difficulties with ADL predicted less emotional/ psychological abuse. Abuser strengths were protective factors with negative correlations to emotional/psy chological abuse, and other contextual factors, including caregiver burden and isolation, were all positively related to emotional/psychological abuse, but not once other variables were statistically controlled.
Hsieh et al., 2009 (31)	Taiwan	Caregivers in nursing homes	Infliction of mental or emotional suffering such as harassment, threats, humiliation, or intimidation of the resident	CPEAB and WSI-C	-	Group intervention using a multi-component approach is necessary for caregivers to help prevent abusive behavior while improving their care-giving knowledge
Wang et al., 2006 (32)	Taiwan	Caregivers	Intentional infliction of mental anguish and involves actions producing fear of violence, isolation or deprivation, or feelings of shame, indignity, and powerless ness.	CPEAB and CBS	Only 2.6% reported never using psychologically abusive behavior during the last 6 months	Young caregivers performed abusive behaviors with increased severity toward elderly recipients. Insufficient preparation for and job burden of heavy caregiving tasks among younger caregivers was found to contribute significantly to the negative aspects of care provision. Female caregivers were more abusive than males.

The results were then grouped in 4 main paragraphs, trying to address the 4 research questions abovementioned.

### 3.1 Definition of psychological abuse

The first research question in this review concerned the presence and rooting of a specific definition given by the authors for psychological violence and abuse, since sharing a single definition is crucial to favor the production of homogeneous scientific literature and data collection.

Unfortunately, the review showed a great variation in the definitions utilized, with three authors using criteria related to questionnaires while seven using simple (but often different and potentially ambiguous) definitions.

In the first cluster, Fulmer et al. (23) considered verbal mistreatment to be present using the verbal aggression subscale of the Conflict Tactics Scale (CTS) (24) and verbal mistreatment groups were created on the basis of scores obtained on the CTS verbal aggression by dichotomizing the sample into a first group reporting no incidents of verbal mistreatment, and a second group reporting at least one incident of verbal mistreatment in the last year.

Similarly, Filipska et al. (25) designed a questionnaire containing questions related to the experience of violence in the last 12 months by the respondents, perpetrators of abuse, exact forms of abuse, reporting the occurrence of the phenomenon to relevant services, and knowledge of victims of violence.

Acierno et al. (26) based their definition of Past-Year Emotional Mistreatment on an affirmative response to any of the following queries in association with the last year: “Has anyone verbally attacked, scolded, or yelled at you so that you felt afraid for your safety, threatened, or intimidated? Has anyone made you feel humiliated or embarrassed by calling you names such as stupid, or telling you that you or your opinion was worthless? Has anyone forcefully or repeatedly asked you to do something so much that you felt harassed or coerced into doing something against your will? Has anyone close to you completely refused to talk to you or ignored you for days at a time, even when you wanted to talk to them?”

The remaining six authors (there were two studies by Conrad et al.) gave more concise definitions: “a form of abuse that involves the use of words, actions, or behaviors that cause psychological harm to an individual” (27); “the intentional infliction of mental anguish and involves actions producing fear of violence, isolation or deprivation, or feelings of shame, indignity, and powerlessness” (28); “the infliction of anguish, pain, or distress through verbal or non-verbal acts” (29–31); “infliction of mental or emotional suffering such as harassment, threats, humiliation, or intimidation of the resident” (32); “abuse that inflicts anguish, pain, or distress through verbal aggression, threats, intimidation, insults, humiliation, and harassment, and can be both intentional or unintentional” (33).

Therefore, the definitions are often partially overlapping, but only in one case do two different studies by different authors share the same definition, provided by the National Center on Elder Abuse (34).

Inconsistency emerges in the choice to distinguish between intentional and unintentional infliction of suffering, with Fulmer et al. (23) being the only authors to clearly focus on failure by the caregiver to satisfy the elder’s basic needs or to protect them from harm. Therefore, Fulmer et al. (23) have approached psychological abuse not only as a proactive infliction of emotional suffering, but also as a lack of proactive protection of older people.

The differences, sometimes minimal, in the definitions used by authors clearly reflect the heterogeneity of the underlying theories hypothesized to date, especially given that most theoretical works have focused exclusively, or mostly, on the physical aspect of violence and abuse. Moreover, most of the theories coexist and, to this day, there has not been a shared theory capable of adequately explaining the whole framework behind the phenomenon of EPA; therefore, it is not surprising that a consensus concerning a definition of EPA has not been reached.

### 3.2 Prevalence

Even though psychological abuse is now considered the most frequent form of elder violence, data regarding the prevalence of the phenomenon are still scarce and fragmented.

The studies included in the review showed a prevalence of psychological abuse of 38% (23), 29% (25), 4.4% (27), 43% (29), 8.4% (26), and 22.6% (33). This shows the significant variability in the prevalence of the phenomenon which, although, undoubtedly reflects the variability in the definitions adopted by the authors (once again highlighting the central issue of uniformity in the definitions).

Moreover, it cannot be ignored that the study setting of the articles included in the review varied significantly. Namely, the article by Conrad et al. (29), which showed the highest prevalence (43%), presented a mixed setting and a self-reporting measurement, creating potential over-reporting biases. On the other hand, the work by Chung et al. (27) showed the lowest prevalence (4.4%) while exploring elder psychological abuse in a community setting, potentially covering the higher number of episodes of abuse that can happen in hospitalized elders.

Fulmer et al. (23), instead, specifically focused only on the theme of verbal mistreatment, potentially leading to an altered prevalence estimation with the exclusion of other potential non-verbal forms of psychological abuse. Furthermore, it should be mentioned that in some of the articles included, such as the one written by Hsieh et al. (32) and the one by Wang et al. (33), the sample under study corresponds to caregivers rather than elders. This point should be considered as an additional factor that could have produced biases in the estimation of the prevalence, as Ho et al. (10) showed that caregivers and third parties are more likely to report abuse than older abused adults.

Additionally, the data presented by Chung et al. (27) and Wang et al. (28, 33) are probably influenced by the practice of filial piety (35–37), meaning filial respect and care for parents, which has been a normative duty and obligation of adult children that can contribute both to reducing the occurrence of elder abuse and to increasing the phenomenon of under-reporting.

Nonetheless, it is also well known that cultural differences radically influence not only the tendency to report cases of EPA but also the very conceptualization of psychological abuse, with the Asian and Asian-American population showing greater tolerance toward potentially abusive situations when compared to Caucasian or African American older adults (38).

### 3.3 Victims’ and perpetrators’ features

Fulmer et al. (23) and Filipska et al. (25) showed that victims of verbal mistreatment were significantly less educated and had reduced income compared to those reporting no verbal mistreatment, while Filipska also found that elder women, respondents over 70 years old, chronically ill and living in the city were more likely to experience psychological abuse. They also found that sons were the most frequent perpetrators (25.9%).

Chung et al. (27) found that emotional abuse was predictive of suicidal ideation and depression, with odds increasing in cases of poor social support. Wang et al. (28) found that younger generations and females were more likely to perform abusive behaviors against elders, while also highlighting the weight of the caregiving burden in the caregivers’ abusive behavior. This finding is supported by the results of Hsieh et al. (32), who found that reducing caregiving burden decreased psychologically abusive behavior, even without significant reduction of work stress.

Liu et al. (31) found that impaired cognition and increased difficulties with activities of daily living (ADL) decreased the rate of emotional abuse, while caregiver burden and isolation increased said risk.

Acierno et al. (26) found that rates of non-reporting of psychological abuse were similar in the case of family/friend-perpetrated abuse (89.9%) and stranger-perpetrated abuse (83.3%), with the reasoning being not wanting to get the perpetrator in trouble and not wanting publicity.

Wang (33) found that individuals with chronic diseases, cognitive impairment and functional dependence showed higher rates of psychological abuse, while also highlighting that in their sample subjects in a domestic setting (vs. institutionalized elders) experienced higher, although not statistically significant, psychological suffering.

### 3.4 Conceptualization and classification

The conceptualization and classification of psychological abuse in older adults appear fragmented across the literature reviewed. Most studies limit themselves to reporting individual items within assessment questionnaires, without offering a comprehensive theoretical framework.

Wang (37) developed the Psychological Elder Abuse Scale (PEAS), a 32-item tool designed for preliminary screening and the identification of psychological abuse.

Filipska et al. (25) employed a questionnaire in which items were grouped into five subcategories of psychological abuse: (1) arrogance and vulgarity, (2) blackmail and threats, (3) closure and isolation, (4) insults and criticism, and (5) mocking.

In another study, Wang et al. (28) designed the Caregiver Psychological Elder Abuse Behavior Scale (CPEAB), consisting of 20 items intended to capture and stratify abusive behaviors enacted by caregivers. This instrument focuses more on caregiver actions than on the impact of abuse on older adults.

Conrad et al. (29) developed a further self-report tool, the Older Adult Psychological Abuse Measure (OAPAM).

Although these instruments are valuable for practical assessment, they fall short of providing a clear conceptual stratification and classification of psychological abuse.

A particularly significant contribution came from Conrad et al. (30), who used concept mapping to identify five conceptual clusters of psychological abuse. These clusters, listed in descending order of severity, are:

- Isolation: identified as the most severe cluster, it includes various forms of social and/or sensory deprivation;
- Threats and intimidation: includes threats of varying intensity. The least severe item is “giving the elder the silent treatment,” while the most severe is “threats of violence.” This cluster is closely associated with both shaming and blaming and insensitivity and disrespect;
- Insensitivity and disrespect: composed of 11 items (ex. “ignoring the effects of pain and illness” and “not allowing the elder to speak for themselves”). Its central position within the conceptual map indicates strong connections with all other clusters. The authors noted that this dimension may capture key characteristics of abusers, with implications for detection, education, prevention, and intervention;
- Shaming and blaming: refers to behaviors that blame or humiliate the elder, including direct verbal attacks such as yelling or swearing;
- Trusted other risk factors: reflects the problematic history of the trusted other and the fear or discomfort experienced by the older adult in relation to them. This cluster is considered useful for identifying potential risk or suspicion of abuse.

These five clusters were further organized into two wider conceptual regions: (1) Physical intimidation, comprising “trusted other risk factors” and “threats and intimidation,” (2) Depersonalization, comprising “isolation,” “insensitivity and disrespect,” and “shaming and blaming.”

This framework not only provides a more precise classification of different manifestations of psychological abuse but also offers a means of understanding their varying degrees of severity.

The findings clearly indicate, therefore, that the scientific community needs a joined effort in trying to reach a shared classification of types of psychological abuse and a shared, valid instrument that could be used to produce comparable data.

In this sense, the works from Filipska et al. (25) and Conrad et al. (30) share a classification that divides psychological abuse into 5 clusters, largely overlapping albeit using different terminology. For example, the clusters of “isolation” and “shaming and blaming” identified by Conrad et al., match, respectively with the subcategories of “closure and isolation” and “insults and criticism” identified by Filipska et al. (25). This finding highlights that authors studying EPA are very close to reaching a univocal classification of the phenomenon, showing a good starting point for future studies.

In summary, the absence of conceptual coherence continues to represent one of the most critical challenges in the field of psychological elder abuse research and assessment.

## 4 Discussion

The narrative review carried out in this paper allowed to answer the research questions that were initially raised.

As for question number 1, “How do definitional variations in elder psychological abuse reflect underlying theoretical and disciplinary perspectives?” the analysis of the 10 articles included in this review revealed that there is no single definition of Elder Psychological Abuse (EPA), with three authors using criteria related to questionnaires and only two papers sharing the same definition.

This issue could be an expression of the lack of significant interest in the topic by the scientific community, which has not yet put in a unanimous effort in the production of a shared definition.

This is also reflected in the fragmentation of the terminology used, sometimes referring to psychological abuse as “verbal” or “emotional.”

In this regard, we believe that a valid baseline could be found in the definition already provided by the National Center on Elder Abuse (“the infliction of distress, pain, or suffering through verbal or nonverbal acts”), to be supplemented with the five subcategories identified by Conrad et al. (30) (isolation, threats and intimidation, insensitivity and disrespect, humiliation and accusations, experiencing distress in relation to the other person’s problematic history) and with part of the definition used by Fulmer et al. (23) that focuses on failure to satisfy elder’s basic needs or to protect older adults from harm, which is more in line with the definition of Health by WHO (39) as a “a state of complete physical, mental and social well-being and not merely the absence of disease or infirmity.”

In addition, the need to define the number of episodes and a possible time frame in which the abuse was perpetrated should be assessed in order to effectively describe the presence of psychological abuse, an essential prerequisite, together with a univocal definition, to allow for the collection of data in a uniform manner at the international level and the consequent informed choice of social and health policies for the prevention of this form of violence.

Regarding the second research question “What patterns emerge in the conceptualization and measurement of elder psychological abuse across different cultural and institutional contexts?,” EPA appears to be the most frequently perpetrated form of abuse today, in contrast to past trends, which saw physical and sexual abuse as more widespread. However, the available data are extremely heterogeneous: the geographical and cultural variability of the studies (because of the impact of filial piety and the tendency to under-report in Asian cultures), methodological differences and the reluctance of victims to report incidents contribute to the absence of unambiguous estimates, as Acierno et al. (26) observed that a very high proportion of cases go unreported (up to 89.9% in the case of family abuse and 83.3% in the case of abuse by strangers), mainly due to fear of retaliation or a desire not to cause issues for the abuser.

A critical issue in the data collection process emerged in the different targets studied in the articles included in the review: Hsieh et al. (32) and Wang et al. (33), in fact, studied samples of caregivers, rather than older adults, which could create a significant bias in the prevalence estimates (10).

Once again, the use of different definitions, scales and questionnaires could’ve undoubtably modified and altered the data collection.

Concerning the third research question “Are there known features of victims and perpetrators of elder psychological abuse?” the review of the studies included revealed certain factors and characteristics associated with victims and others with abusers. Among the former, the literature most frequently reports low level of education, female gender, the presence of chronic diseases, social isolation, reduced autonomy in both daily activities and financial management and, in general, a vulnerable home environment.

As for abusers, characteristics associated with a greater tendency to perpetrate psychological abuse against older adults have been identified in young age, female gender, family relationship (particularly direct descendants, such as sons), and the presence of unfavorable psychological conditions, such as caregiver burden, which seems to support the caregiver stress theory.

Concerning the 4^th^ question “How do current conceptualization and classification explain the complexity and heterogeneity observed in elder psychological abuse research?,” the review also highlights how the literature does not offer universally valid tools for classifying and assessing this form of violence. Most of the studies analyzed used questionnaires aimed at caregivers or victims, tools that are mainly descriptive but lack a solid conceptual framework and are susceptible to the subjectivity of the person responding to the questionnaire.

In this context, the most structured contribution was proposed by Conrad et al. (30), who divided EPA into two macro-categories further divided into five clusters, offering a very detailed view of psychological violence that could help the international scientific community deepen its knowledge of this worrying phenomenon.

Specifically, the subdivision into the five clusters of isolation, threats and intimidation, insensitivity and disrespect, shaming and blaming and trusted other risk factors appears to provide an in-depth description of psychological abuse that, first of all, acknowledges and legitimates various forms of abuse which are often underestimated and undervalued.

This classification greatly overlaps with the one provided by Filipska et al. (25), showing that the scientific community has almost reached a consensus in the subdivision of EPA and this could be a stepping stone for future research.

The contribution by Conrad et al. was also highly valuable in the development, testing and validation of a self-report tool, the Older Adult Psychological Abuse Measure (OAPAM) (29), which can be utilized by both researchers and clinicians, providing a tool that could be essential in the process of uniformization of data collection.

This process is crucial to deepen the knowledge in the field of EPA, because of the consequences for victims: Chung et al. (27) have shown that psychological abuse is predictive of suicidal ideation and depression, with a significant increase in risk in the presence of poor social support, while Yunus et al. (40) highlighted that among the subtypes of elder abuse, psychological abuse and neglect were found to significantly decrease the quality of sleep, while other subtypes did not.

Furthermore, Wang et al. (28) observed that older adults living in domestic settings, compared to those in institutional settings, experience higher levels of psychological distress, although this finding did not reach statistical significance.

Another finding of this review was that the first article that met the inclusion criteria was published only in 2006, clearly showing how the issue of EPA has been only recently studied as a specific form of abuse itself, while the low number of studies included highlights how poor the current knowledge is in this field, which has received little attention over the years.

Ultimately, this narrative review has demonstrated that despite the high prevalence of EPA, albeit highly variable depending on the cultural and geographical context, it has received little attention from the international medical scientific community over the years, despite the severe consequences that this form of violence can have on the health of older people.

This shows that although EPA is often associated with other forms of abuse and therefore studied in relation to them, it is a phenomenon that has been little studied in its own right, which may reduce our understanding of its specific nuances and mechanisms.

The findings have also shown the high heterogeneity in the definitions and classification, even though there seem to be valid enough starting points for future unification of complete conceptualization of EPA.

The results of the review indicate some potential areas of risk and attention for policymakers regarding both victims and abusers.

Concerning the reduction of risk for victims, results suggest the necessity to provide investments in constant education for all ages and in protection of females and isolated and chronically ill older adults.

As for potential abusers, data indicate that the target of policymakers should also be younger adults (especially sons) and caregivers both in community and hospital settings, to maximize the potential for prevention while also trying to favor the growth of a culture of aid and respect for older adults.

Moreover, the data presented by Acierno et al. (26) highlighted the need to provide increased support to older adults in reporting the psychological abuse they have suffered, reducing feelings of shame and publicity and assuring full protection after pressing charges.

Finally, concerning limitations of this review, the choice to use a single search database (PubMed), although considered by the authors to be the most popular and comprehensive, may not have shown all studies published on this topic and published in nonmedical search engines.

However, this choice was made in the broader context of the authors’ academic background, which is rooted primarily in medicine and, more specifically, in legal and forensic medicine, leading to the decision to limit the search to the medical field and not to delve into areas unfamiliar to the authors, such as psychology, sociology and ecology.

The academic and geographical background of the authors may also be responsible for potential biases in the interpretation of the data extracted in this narrative review, especially concerning the lack of knowledge in the fields of psychology and sociology, which the authors hope to integrate in further studies with a multidisciplinary approach.

The keywords chosen may have also excluded articles concerning the broader context of “adult safeguarding” and “adult protection,” search terms that are used in countries such as the UK, limiting the number of studies yielded by the research.

## 5 Conclusion

Elder abuse is a substantial global public health and human rights problem, with psychological abuse being, albeit the most common, certainly one of the lesser known and studied forms of violence, the impact of which must not be overlooked. The review conducted in this paper showed that the available literature on Elder Psychological Abuse is still limited and fragmented, with a small number of studies dedicated to this specific topic, the absence of a shared definition and the lack of standardized categorization and assessment tools, with an extreme variability in the prevalence of the phenomenon, which probably reflects the presence of different definitions, socio-cultural heterogeneity and reduced reporting rates. The results clearly show the necessity of deepening knowledge concerning this issue and mainly for the effort of the scientific community to produce a shared definition that can help produce homogeneous international data to promote social and health protocols investing in education, growth of a culture of aid and respect of older adults and protection of fragile categories such as females, isolated and chronically ill older adults, both in community and hospital settings.

## Data Availability

The raw data supporting the conclusions of this article will be made available by the authors, without undue reservation.

## References

[1] Organisation mondiale de la santé. Rapport mondial sur la violence et la santé. Genève: Organisation mondiale de la santé (2002).

[2] Casella C Aquino CI Sarno L Di Donna G Capasso E. Is there a lesser value type of violence? Older people abuse: “the silence of the lambs”. Legal Med. (2024) 66:102359. doi: 10.1016/j.legalmed.2023.102359, PMID: 38035529

[3] Bugelli V Campobasso CP Feola A Tarozzi I Abbruzzese A Di Paolo M. Accidental injury or “shaken elderly syndrome”? Insights from a case report. Health. (2023) 11:228. doi: 10.3390/healthcare11020228, PMID: 36673596 PMC9859477

[4] World Health Organization. World report on ageing and health [internet]. Geneva: World Health Organization (2015).

[5] Penhale B. Bruises on the soul: older women, domestic violence and elder abuse. Bold. (1998) 8:16–30. 12295470

[6] Roberto KA McPherson MC Brossoie N. Intimate partner violence in late life: a review of the empirical literature. Violence Against Women. (2013) 19:1538–58. doi: 10.1177/1077801213517564, PMID: 24476758 PMC10225065

[7] Aquino CI Marisei M Surico D Di Donna G. “Short epic” on gender-based violence in the healthcare landscape, according to Italian legislation: an example of modern social and cultural evolution. Ital J Gynaecol Obstet. (2024) 36:130. doi: 10.36129/jog.2023.126

[8] Di Donna G Di Lorenzo P Aquino CI Marisei M Casella C Surico D . Gender violence during the three ages of life and the impact of the Covid-19 pandemic: a review. Int J Social Determinants Health Health Services. (2024) 54:423–35. doi: 10.1177/27551938241247776, PMID: 38646684

[9] Sánchez Salgado CD. Features of elder abuse and mistreatment in Puerto Rico. PR Health Sci J. (2007) 26:35–41. 17674872

[10] Ho CS Wong SY Chiu MM Ho RC. Global prevalence of elder abuse: a meta-analysis and meta-regression. East Asian Arch Psychiatr. (2017) 27:43–55. 28652497

[11] Pillemer KA Wolf RS (Eds.). Elder abuse: Conflict in the family. New York, NY, England: Auburn House Publishing Co (1986). 356 p.

[12] Wilber KH McNeilly DP. Elder abuse and victimization In: Handbook of the psychology of aging. 5th ed. Birren JE Schaie KW editors. San Diego, CA, US: Academic Press (2001). 569–91.

[13] Burnight K Mosqueda L. Theoretical model development in elder mistreatment. National Criminal Justice Reference Service; (2011). Available online at: https://www.ncjrs.gov/pdffiles1/nij/grants/234488.pdf (Accessed May 07, 2025).

[14] Mosqueda L Burnight K Gironda MW Moore AA Robinson J Olsen B. The abuse intervention model: a pragmatic approach to intervention for elder mistreatment. J Am Geriatr Soc. (2016) 64:1879–83. doi: 10.1111/jgs.14266, PMID: 27550723 PMC5026887

[15] Abolfathi Momtaz Y Hamid TA Ibrahim R. Theories and measures of elder abuse. Psychogeriatrics. (2013) 13:182–8. doi: 10.1111/psyg.12009, PMID: 25913768

[16] World Health Organisation (2018). The ecological framework. Available online at: http://www.who.int/violenceprevention/approach/ecology/en/ (Accessed May 07, 2025).

[17] Fundinho JF Pereira DC Ferreira-Alves J. Theoretical approaches to elder abuse: a systematic review of the empirical evidence. JAP. (2021) 23:370–83. doi: 10.1108/JAP-04-2021-0014

[18] National Research Council (US) Panel to Review Risk and Prevalence of Elder Abuse and Neglect In: Bonnie RJ Wallace RB, editors. Elder mistreatment: Abuse, neglect, and exploitation in an aging America. Washington (DC): National Academies Press (US) (2003) 22812026

[19] Sukhera J. Narrative reviews in medical education: key steps for researchers. J Grad Med Educ. (2022) 14:418–9. doi: 10.4300/JGME-D-22-00481.1, PMID: 35991097 PMC9380643

[20] Ferrari R. Writing narrative style literature reviews. Med Writ. (2015) 24:230–5. doi: 10.1179/2047480615Z.000000000329

[21] Grant MJ Booth A. A typology of reviews: an analysis of 14 review types and associated methodologies. Health Inf Libr J. (2009) 26:91–108. doi: 10.1111/j.1471-1842.2009.00848.x, PMID: 19490148

[22] Tricco AC Lillie E Zarin W O’Brien KK Colquhoun H Levac D . PRISMA extension for scoping reviews (PRISMA-ScR): checklist and explanation. Ann Intern Med. (2018) 169:467–73. doi: 10.7326/M18-0850 30178033

[23] Fulmer T Rodgers RF Pelger A. Verbal mistreatment of the elderly. J Elder Abuse Negl. (2014) 26:351–64. doi: 10.1080/08946566.2013.801817, PMID: 24910894 PMC4051298

[24] Touliatos J Perlmutter BF. Handbook of family measurement techniques. Thousand Oaks, Calif: Sage (2001).

[25] Filipska K Biercewicz M Wiśniewski A Kędziora-Kornatowska K Ślusarz R. Prevalence and associated factors of elder psychological abuse- a cross- sectional screening study, based on a hospitalized community from Poland. Arch Gerontol Geriatr. (2020) 90:104152. doi: 10.1016/j.archger.2020.104152, PMID: 32623311

[26] Acierno R Steedley M Hernandez-Tejada MA Frook G Watkins J Muzzy W. Relevance of perpetrator identity to reporting elder financial and emotional mistreatment. J Appl Gerontol. (2020) 39:221–5. doi: 10.1177/0733464818771208, PMID: 29703128

[27] Chung J Jeon E Park TW Park J Chung S. Emotional abuse and depression as factors associated with suicidal ideation in community-dwelling older adults: mediation analysis. Psychogeriatrics. (2025) 25:e70024. doi: 10.1111/psyg.70024, PMID: 40090698

[28] Wang JJ. Psychological abuse and its characteristic correlates among elderly Taiwanese. Arch Gerontol Geriatr. (2006) 42:307–18. doi: 10.1016/j.archger.2005.08.006, PMID: 16214246

[29] Conrad KJ Iris M Ridings JW Langley K Anetzberger GJ. Self-report measure of psychological abuse of older adults. Gerontologist. (2011) 51:354–66. doi: 10.1093/geront/gnq103, PMID: 21173437

[30] Conrad KJ Iris M Ridings JW Rosen A Fairman KP Anetzberger GJ. Conceptual model and map of psychological abuse of older adults. J Elder Abuse Negl. (2011) 23:147–68. doi: 10.1080/08946566.2011.558784, PMID: 21462048

[31] Liu PJ Conrad KJ Beach SR Iris M Schiamberg LB. The importance of investigating abuser characteristics in elder emotional/psychological abuse: results from adult protective services data. J Gerontol B Psychol Sci Soc Sci. (2019) 74:897–907. doi: 10.1093/geronb/gbx064, PMID: 28521064

[32] Hsieh HF Wang JJ Yen M Liu TT. Educational support group in changing caregivers’ psychological elder abuse behavior toward caring for institutionalized elders. Adv Health Sci Educ Theory Pract. (2009) 14:377–86. doi: 10.1007/s10459-008-9122-6, PMID: 18516696

[33] Wang JJ Lin JN Lee FP. Psychologically abusive behavior by those caring for the elderly in a domestic context. Geriatr Nurs. (2006) 27:284–91. doi: 10.1016/j.gerinurse.2006.08.016, PMID: 17045127

[34] National Center on Elder Abuse. (2003). The basics: major types of abuse. Available online at: http://www.elderabusecenter.org/default.cfm?p=basics.cfm (Accessed on February 18, 2007)

[35] Sung KT. An exploration of actions of filial piety. J Aging Stud. (1998) 12:369–86. doi: 10.1016/S0890-4065(98)90025-1

[36] Fu YY Guo Y Bai X Chui EWT. Factors associated with older people’s long-term care needs: a case study adopting the expanded version of the Anderson model in China. BMC Geriatr. (2017) 17:38. doi: 10.1186/s12877-017-0436-1, PMID: 28143442 PMC5282820

[37] Zang Z. The care types choice in filial culture: a cross-sectional study of disabled elderly in China. Front Public Health. (2022) 10:954035. doi: 10.3389/fpubh.2022.954035, PMID: 36148366 PMC9485573

[38] Moon A. Perceptions of elder abuse among various cultural groups: similarities and differences. Generations. (2000) 24:75–80.

[39] Schramme T. Health as complete well-being: the WHO definition and beyond. Public Health Ethics. (2023) 16:210–8. doi: 10.1093/phe/phad017, PMID: 38333767 PMC10849326

[40] Yunus RM Hairi NN Yuen C Sooryanarayana R Hairi F Ismail N . Does abuse in late life worsen sleep quality? A two-year prospective cohort study among rural older adults. Int J Geriatr Psychiatry. (2019) 34:60–6. doi: 10.1002/gps.4986, PMID: 30230023

